# FANC Pathway Promotes UV-Induced Stalled Replication Forks Recovery by Acting Both Upstream and Downstream Polη and Rev1

**DOI:** 10.1371/journal.pone.0053693

**Published:** 2013-01-24

**Authors:** Emilie Renaud, Filippo Rosselli

**Affiliations:** 1 Université Paris Sud, UMR8200, « Equipe Labellisée Ligue Contre le Cancer », Institut de Cancérologie Gustave-Roussy, Villejuif, France; 2 CNRS, UMR8200, « Equipe Labellisée Ligue Contre le Cancer », Institut de Cancérologie Gustave-Roussy, Villejuif, France; University of Massachusetts Medical School, United States of America

## Abstract

To cope with ultraviolet C (UVC)-stalled replication forks and restart DNA synthesis, cells either undergo DNA translesion synthesis (TLS) by specialised DNA polymerases or tolerate the lesions using homologous recombination (HR)-based mechanisms. To gain insight into how cells manage UVC-induced stalled replication forks, we analysed the molecular crosstalk between the TLS DNA polymerases Polη and Rev1, the double-strand break repair (DSB)-associated protein MDC1 and the FANC pathway. We describe three novel functional interactions that occur in response to UVC-induced DNA lesions. First, Polη and Rev1, whose optimal expression and/or relocalisation depend on the FANC core complex, act upstream of FANCD2 and are required for the proper relocalisation of monoubiquitinylated FANCD2 (Ub-FANCD2) to subnuclear foci. Second, during S-phase, Ub-FANCD2 and MDC1 relocalise to UVC-damaged nuclear areas or foci simultaneously but independently of each other. Third, Ub-FANCD2 and MDC1 are independently required for optimal BRCA1 relocalisation. While RPA32 phosphorylation (p-RPA32) and RPA foci formation were reduced in parallel with increasing levels of H2AX phosphorylation and MDC1 foci in UVC-irradiated FANC pathway-depleted cells, MDC1 depletion was associated with increased UVC-induced Ub-FANCD2 and FANCD2 foci as well as p-RPA32 levels and p-RPA32 foci. On the basis of the previous observations, we propose that the FANC pathway participates in the rescue of UVC-stalled replication forks in association with TLS by maintaining the integrity of ssDNA regions and by preserving genome stability and preventing the formation of DSBs, the resolution of which would require the intervention of MDC1.

## Introduction

DNA damage is a primary source of cellular stress and a leading cause of cancer [1]. To cope with DNA lesions, cells have developed an integrated and tightly regulated molecular network called the DNA damage response (DDR), in which cell cycle checkpoints and DNA repair pathways collaborate to efficiently restore the integrity of the genetic material [2]. To avoid the induction and fixation of mutations and to avoid the transmission of genetic modifications, DNA lesions must be eliminated before DNA replication. Nevertheless, replication forks will inevitably encounter DNA lesions and stall. To restore DNA synthesis and permit cells to progress into mitosis, cells exploit DNA damage tolerance (DDT) pathways that involve either translesion synthesis (TLS) by specialised DNA polymerases or using homologous recombination (HR)-based mechanisms, such as template switching (TS) and break-induced replication (BIR) [3], [4].

DNA damage induced by ultraviolet C radiation (UVC) is a well-characterised roadblock for ongoing replication forks. UVC induces two major DNA lesions, cyclobutane pyrimidine dimers (CPDs) and 6-4 pyrimidine-pyrimidone photoproducts (6,4-PPs). These lesions are primarily removed through the error-free nucleotide excision repair (NER) pathway [2]. Germline recessive mutations that lead to NER defects are responsible for the classic form of the skin cancer predisposition syndrome xeroderma pigmentosum (XP). The products of the seven cloned *XP* genes (*XP-A* to *XP-G*) work sequentially to remove UVC-induced DNA lesions. Inactivation of any of these genes is associated with cellular hypersensitivity to UVC, increased UV-induced mutagenesis and inefficient removal of both CPDs and 6,4-PPs [5].

Because unrepaired UVC-induced lesions halt the ongoing progression of replication forks, they trigger RAD6/RAD18-mediated monoubiquitinylation of PCNA followed by the assembly of TLS polymerases, including Polη, which has been implicated in the error-free bypass of UVC-induced CPDs [6], [7]. Bi-allelic hereditary mutations in the Polη coding gene *POLH* have been identified as the molecular defect underlying the skin cancer predisposition syndrome XP-variant (XP-V) [8]. Compared to classically XP-affected individuals, XP-V patients' photosensitivity is reduced and skin cancers develop later. XP-V cells repair UVC-induced lesions at a normal rate and display modest increase in sensitivity to UVC exposure. However, these cells are unable to replicate past UVC lesions. Therefore, XP-V cells accumulate mutations and small deletions [9], [10], contributing to the cancer predisposition associated with XP-V.

UVC exposure also activates the FANC pathway, which is involved in safeguarding DNA replication and cell division in both unstressed and DNA-damaged cells [11]–[13]. Bi-allelic germline mutations in any of at least 15 *FANC* genes (*FANCA* to *FANCP*) lead to Fanconi anemia (FA), which is characterised by bone marrow failure, developmental defects, cancer predisposition, cell cycle abnormalities and chromosomal instability [14], [15]. The FANC pathway is generally activated in response to replicative stress. Loss of function of this pathway is primarily associated with cellular and chromosomal hypersensitivity to DNA interstrand crosslinks (ICLs). In response to ICLs, the FANC pathway directly or functionally interacts with several other DDR/DDT factors, including the checkpoint proteins ATR and CHK1, the DSB repair and recombination proteins MRE11/RAD50/nibrin, H2AX, BRCA1, BLM, RAD51, PCNA, the single-stranded DNA (ssDNA)-binding protein RPA and the TLS polymerase Rev1 [16]–[30].

Finally, recent works have demonstrated a role for MDC1 in response to either UV exposure [31] or to replication stress [32]. MDC1 was first described as a large mediator protein recruited at DSB sites by γH2AX [33]. This protein is necessary for the RNF8- and RNF168-mediated histones post-traductional modifications that are crucial for the recruitment of different proteins to DNA damage sites, including BRCA1 and 53BP1 [33]. However, the function of MDC1 in response to UV-induced stalled replication forks remains poorly characterized. Nevertheless, similarly to Polη-deficiency, siRNA-mediated MDC1 downregulation does not modify DNA repair capabilities while increasing only marginally the cellular sensitivity to UV [31].

In this study, we analysed the specific crosstalk that occurs among NER proteins, the TLS DNA polymerases Polη and Rev1, the DSB-associated protein MDC1 and the FANC pathway in response to UVC. Based on our observations, we propose a working model of how the TLS and FANC pathways collaborate to permit cells to recover from stalled replication forks and to maintain genetic stability.

## Results

### FANCC depletion affects cellular resistance to UVC

**Figure 1:**
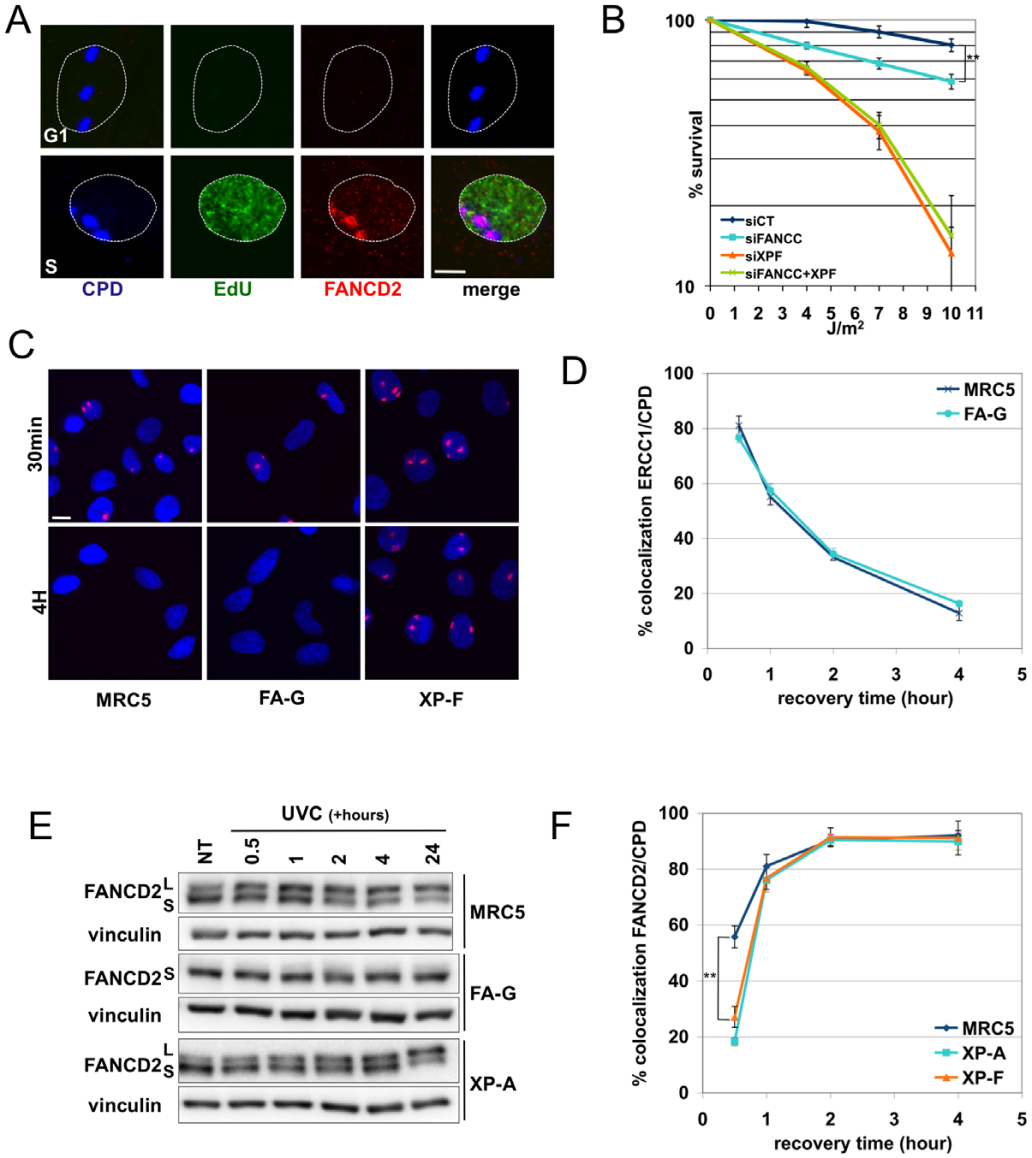
UV irradiation activates the FANC pathway in S-phase and independently of the Nucleotide Excision Repair pathway. (**A**) FANCD2 localization in primary fibroblasts. Wt primary fibroblasts (AS977) were synchronized in G1 by serum starvation, diluted and released in S-phase. Cells were locally UVC-irradiated (100 J/m^2^) and fixed 6 h later. EdU was added to the medium (10 µM) for 10 min before fixation. Represented merge: FANCD2 (red)/EdU (green)/CPD (blue). Bar: 10 µm. (**B**) Clonogenic survival of UVC-irradiated cells. HeLa cells were seeded 48 h after transfection with either a nontargeted siRNA (CT) or with siRNA targeting FANCC, XPF or XPF and FANCC. They were mock- or UVC-irradiated 6 h later. Surviving colonies were fixed and counted 10 days later. Each point on survival curves represents the mean of 3 independent experiments. ** indicates a significant statistical difference (p<0,01) as calculated using a T-student test. (**C**) 6,4-PPs removal in MRC5, FA-G (PD352) and XP-F (GM8437) cells. Cells were locally UVC-irradiated (100 J/m^2^) and fixed 30 min or 4 h later. Representative merge: 6,4-PP antibody (red)/DNA (blue, DAPI). Bar: 20 µm. (**D**) Recruitment kinetics of ERCC1. MRC5 and FA-G cells were locally UVC-irradiated (100 J/m^2^) and fixed at different time points. BrdU was added to the medium (10 µM) for 10 min before fixation. Co-localization between ERCC1 and CPDs was quantified in BrdU-positive cells. At least 100 cells per time point were scored. Each data point represents the mean of three independent experiments. (**E**) FANCD2 monoubiquitinylation analysis. MRC5, FA-G, and XP-A fibroblasts were globally UVC-irradiated (10 J/m^2^) and harvested at the indicated times. (**F**) Recruitment kinetics of FANCD2 in MRC5, XP-A (XP12RO) and XP-F (GM8437) cells. Cells were treated as in (D). Each data point represents the mean of three independent experiments. ** indicates a significant statistical difference (p<0,01) as calculated using a T-student test.

The presence of DNA repair proteins on UVC-induced DNA lesions *in vivo* was efficiently analysed by immunofluorescence following local irradiation of cells at 100 J/m^2^. Nuclear local irradiated regions (LIR) were easily visualised through the use of specific antibodies directed against CPDs or 6,4-PPs (Figure 1A and 1C). By co-staining cells with a DNA replication tracker (BrdU or EdU), an anti-UVC-induced lesion and/or an anti-FANCD2 antibody, we observed that FANCD2 was recruited to LIR only in replicative and post-replicative primary or transformed cells (Figures 1A and S1A). This contrasts with the well-known response of NER proteins, which rapidly relocalise to damaged LIR independently of the cell cycle phase (Figures S1C and S1D). These observations indicate that FANCD2 redistribution to damaged nuclear areas, a well-known outcome FANC pathway activation, is associated with DNA replication difficulties and not simply with DNA repair events.

We sought to understand the functional meaning of the activation of the FANC pathway after UVC exposure. We first measured cell survival in response to UVC in DDR/DDT-proficient cells and in FANC pathway- or NER-depleted cells by measuring the clonogenicity of isogenic HeLa cells transfected with siRNAs targeting *FANCC* and/or *XPF* to inactivate the FANC and NER pathways, respectively. Compared to the parental HeLa cells (LD_50_>20 J/m^2^), NER deficiency caused UVC hypersensitivity (LD_50_≅ 5 J/m^2^), whereas FANC pathway loss-of-function caused a modest but statistically significant (p<0.01) increase in cell sensitivity to UVC (LD_50_≅12 J/m^2^) (Figure 1B). Similar results were obtained after down-regulating FANCD2 expression (data not shown). Thus, the FANC pathway appears either to contribute to the prevention of the death of UVC-challenged cells or to sustain their active proliferation following irradiation, preventing permanent growth arrest and senescence.

### The FANC and NER pathways function independently of each other

To determine if the increased sensitivity to UVC following FANC pathway inactivation is associated with alterations in DNA repair, we monitored the disappearance of 6,4-PPs and CPDs from LIR in DDR-proficient and in NER- and FANC-deficient cells (Figures 1C, S1B and data not shown). In DNA repair-proficient and FANC pathway-deficient cells, the elimination of the two photoproducts followed the same kinetics (Figure S1B). 6,4-PPs were eliminated after 4 h, whereas CPDs persisted longer: approximately 75% of the initially induced lesions were eliminated by 48 h post-irradiation. In sharp contrast, in NER-deficient cells, both lesions were still largely detectable 48 h after UVC exposure (Figure S1B and data not shown). Moreover, no significant difference in the redistribution of ERCC1, an NER protein, to LIR was observed between FANC-deficient and FANC–proficient cells (Figures 1D, S1C and S1D), suggesting that the NER pathway operates independently of the FANC pathway. For reasons that are yet unclear, NER proficiency is necessary for rapid monoubiquitinylation and relocalisation of FANCD2 to damaged DNA (Figures 1E, S1E and 1F). However, by 1 h post-treatment, FANCD2 was efficiently relocalised to LIR in both NER-deficient and -proficient cells. The level of monoubiquitinylated FANCD2 in NER-deficient cells was similar to that in NER-proficient cells by 2 h post-UVC and was clearly more elevated at 24 h (Figure S1E), most likely due to the long-term persistence of DNA lesions in the DNA repair-deficient cells.

### UVC irradiation independently activates the FANC pathway and MDC1

The DSB repair-associated protein MDC1 was recently shown to be among the DDR proteins that are activated by UVC in an ATR-dependent manner [31], as is FANCD2 [16]. As we demonstrated above in FANC-deficient cells, MDC1-depleted cells relocalise NER proteins to LIR normally and are fully proficient in the removal of UVC lesions [31]. In that study, MDC1 accumulated in LIR with kinetics similar to that observed here for FANCD2. Compared to FANCD2, MDC1 relocation exhibited a more pronounced delay in NER-deficient cells, most likely because MDC1 also relocalises in a NER-dependent manner during G1-phase (Figure S2A and [31]). However, NER-proficient and NER-deficient cells recovered similar levels of MDC1 foci by 24 h post-irradiation (data not shown). These observations led us to investigate a potential functional link between MDC1 and FANCD2.

**Figure 2:**
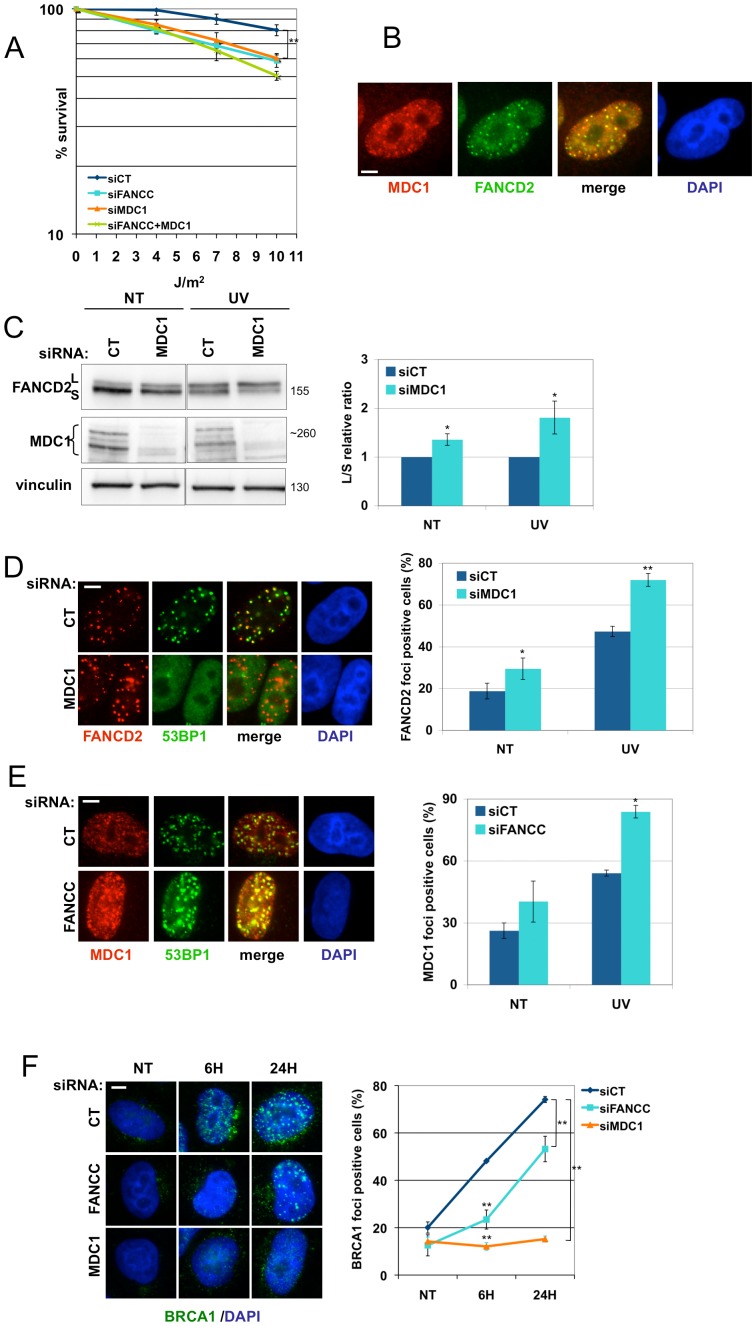
FANCD2 and MDC1 independently regulate BRCA1 recruitment. (**A**) Clonogenic survival of UVC-irradiated cells. HeLa cells were seeded 48 h after transfection with indicated siRNAs and were mock- or UVC-irradiated 6 h later. Surviving colonies were fixed and counted 10 days later. Each data point represents the mean of three independent experiments. ** indicates a significant statistical difference (p<0,01) as calculated using a T-student test. (**B**) MDC1 (red)/FANCD2 (green) co-localization analysis 24 h after UVC irradiation (10 J/m^2^) in HeLa cells. Bar: 5 µm. (**C**) FANCD2 monoubiquitinylation analysis. HeLa cells were mock- or UVC-irradiated (10 J/m^2^) 48 h after transfection and harvested 4 h later. The lysates were analyzed by Western blotting using the indicated antibodies. The Long form (monoubiquitinylnated form)/Short form relative ratio (L/S ratio) was measured using Image J software. Each data point represents the mean of three independent experiments. * indicates a significant statistical difference (p<0,05) as calculated using a T-student test. (**D**) and (**E**) Immunofluorescence analysis of FANCD2/53BP1 and MDC1/53BP1 focus formation. HeLa cells were mock- or UVC-irradiated (10 J/m^2^) 48 h after transfection with untargeted or a FANCC-targeted siRNA. 24 h later, they were prepermeabilized then fixed. For foci-positive cell quantification, cells with more than 5 foci were counted. Bar: 5 µm. Each data point represents the mean of three independent experiments. * and ** indicate a significant statistical difference (p<0,05 and p<0,01, respectively) as calculated using a T-student test. (**F**) BRCA1 relocalization to UVC-induced subnuclear foci. Hela cells were transfected with untargeted, FANCC- or MDC1-targeted siRNA, processed as in (D) and fixed 4 and 24 h later. For foci-positive cell quantification, cells with more than 5 foci were counted. Bar: 5 µm. Each data point represents the mean of three independent experiments. ** indicates a significant statistical difference (p<0,01) as calculated using a T-student test.

While individual depletion of FANCC or MDC1 in HeLa cells induced a modest but significant (p<0.01) increase in UVC cytotoxicity (Figures 1B and 2A), simultaneous depletion of FANCC and MDC1 did not modify the cytotoxic effects observed in singly depleted cells (Figure 2A). Moreover, following a global irradiation protocol that permits a more refined analysis of protein relocalisation to DNA lesions, we observed a consistent co-localisation of FANCD2 and MDC1 foci (Figure 2B).

In both non-irradiated and UVC-irradiated cells, MDC1 depletion by siRNA induced a consistent increase in FANCD2 monoubiquitinylation (p<0.05; Figures 2C and S2B). Accordingly, the frequency of cells showing FANCD2 foci and the number of foci per cell increased in MDC1-depleted cells (Figures 2D and S2C). Similar results were obtained in U2OS cells (Figure S5A and S5B). Cells with loss-of-function of the FANC pathway (FA patient-derived cell lines or HeLa cells with siRNA-mediated depletion of FANCC) exhibited a strong accumulation, persistence and brightness of UVC-induced MDC1 foci (Figures 2E and S2D).

Altogether, the data presented here strongly support the hypothesis that MDC1 and the FANC pathway, although activated independently of each other, operate to assure the optimal function of common downstream event(s) during the replication of UVC-damaged DNA. Interestingly, the well-known tumour suppressor and DSB repair protein BRCA1, whose optimal relocalisation to subnuclear foci at DNA lesions requires MDC1 (in the presence of DSBs) and a proficient FANC pathway (in response to ICLs), was recently shown to be involved in the response of cells to UVC [19], [34]. Consequently, we decided to explore BRCA1 relocalisation to UVC-induced nuclear foci in FANC pathway- and MDC1-depleted cells. siRNA-mediated depletion of FANCC in HeLa cells significantly delayed BRCA1 recruitment to UV-induced foci after irradiation, whereas MDC1 depletion impeded the assembly of BRCA1 foci (Figure 2F). Thus, optimal BRCA1 relocalisation following UVC appears to require both a functional FANC pathway and MDC1.

The observations presented in this section suggest that the mutually independent activation and relocalisation of MDC1 and FANCD2 to UVC-damaged nuclear areas are involved in BRCA1 relocalisation, which could participate in both stalled fork recovery and DSB repair.

### UVC activation of the FANC pathway prevents DSB formation and favours the formation and maintenance of ssDNA regions downstream of UVC-stalled forks

UVC exposure induces post-translational modifications of several proteins, including PCNA, which is monoubiquitinylated (Ub-PCNA) in a RAD6/RAD18-dependent manner [35], and RPA32 and H2AX, which are phosphorylated in an ATR-dependent manner [36], [37]. To better define the role of the FANC pathway and MDC1 in UVC responses, we characterised the post-translational modifications of PCNA, RPA32 and H2AX.

**Figure 3:**
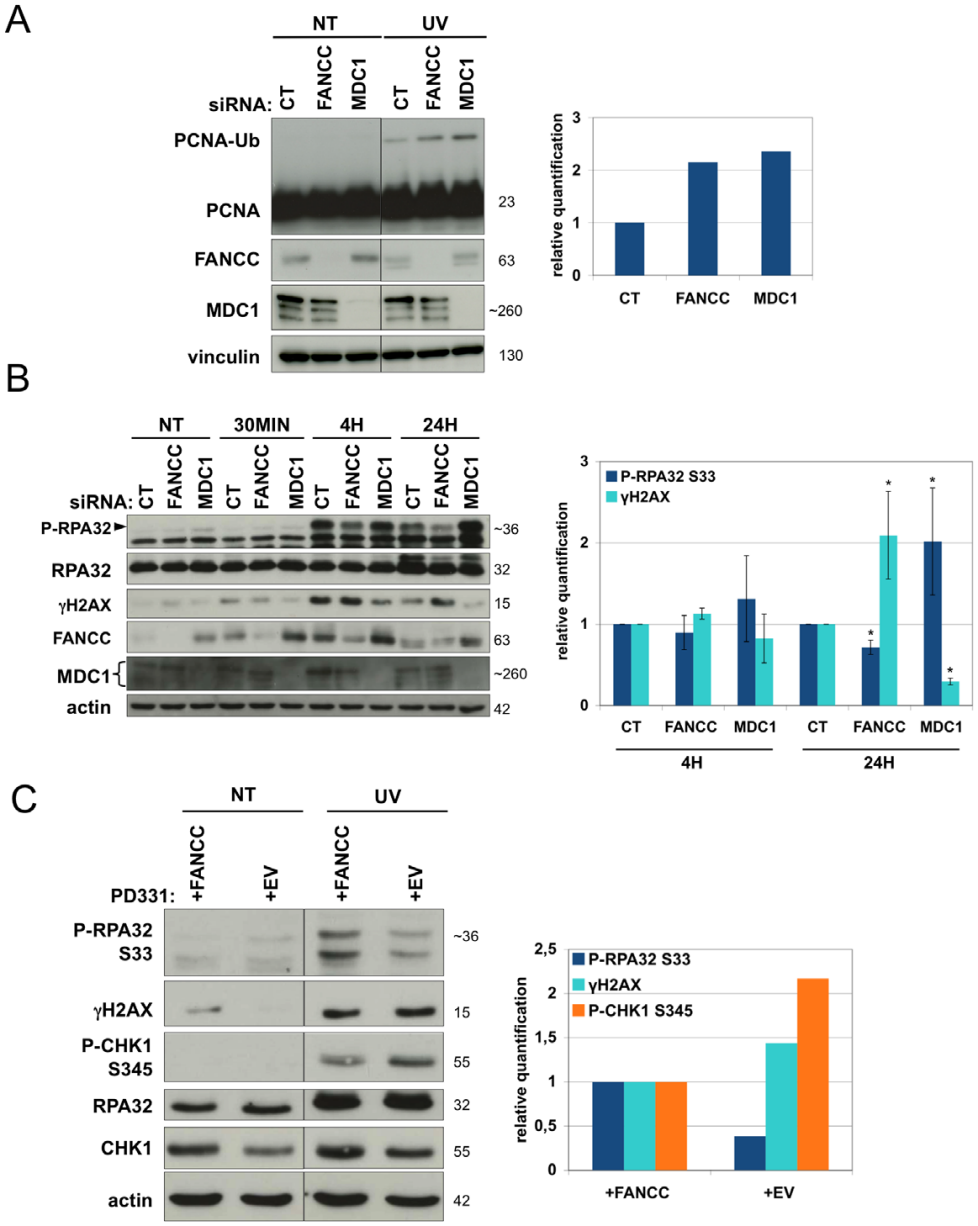
FANC pathway prevents DSB formation and favors the maintenance of ssDNA regions. (**A**) PCNA monoubiquitinylation and (**B**) RPA32 and H2AX phosphorylation analysis. HeLa cells were mock- or UVC-irradiated (10 J/m^2^) 48 h after transfection and harvested 24 h later (A) or at the indicated times (B). The lysates were analyzed by Western blotting using the indicated antibodies. Protein monoubiquitinylation (A) and phosphorylation (p-RPA32/total RPA and γH2AX/actin; B) were quantified using Image J software. Each data point represents the mean of two (A) or three (B) independent experiments. * indicates a significant statistical difference (p<0,05) as calculated using a T-student test. (**C**) RPA32, CHK1 and H2AX phosphorylation quantification. PD331 (+EV or +FANCC) cells were mock- or UVC-irradiated (10 J/m^2^) and harvested 24 h later. The lysates were analyzed by Western blotting using the indicated antibodies. Phosphorylation quantification (p-RPA32/total RPA, p-CHK1/total CHK1 and γH2AX/actin) was performed using Image J software. Each data point represents the mean of two independent experiments.

Ub-PCNA is associated with the induction and persistence of stalled replication forks. Depletion of FANCC or MDC1 resulted in similarly increased levels of Ub-PCNA in response to UVC exposure, indicating greater accumulation of unresolved stalled forks (Figure 3A). This result is consistent with previous work describing the UVC response in BRCA1-deficient cells [34] and supports the involvement of BRCA1 in the recovery of stalled replication forks downstream of FANC and MDC1.

The presence of phophorylated RPA32 (p-RPA2) is associated with stretches of ssDNA at stalled forks. These stretches of ssDNA occur as a consequence of decoupling between the replicative DNA polymerase and the replicative DNA helicase. The presence of p-H2AX (γH2AX) indicates the presence of DSBs or, more generally, altered chromatin status. We observed consistent differences (p<0.05) in the phosphorylation and/or nuclear relocalisation of RPA and H2AX in wild-type (wt), FANC pathway- depleted/deficient and MDC1-depleted cells (Figure 3B, 3C and S3A). In wt cells, p-RPA32 was detected 4 h post-UVC exposure and persisted for at least 24 h (Figure 3B). Based on the observed kinetics of ERCC1 recruitment (Figure S1E) and previously published data [38], the accumulation of p-RPA32 in wt cells is clearly independent of the DNA repair process and is instead likely to be associated with replication issues. FANC pathway downregulation or deficiency was correlated with decreased levels of p-RPA32, with decreased frequency of p-RPA32 foci and with enhanced γH2AX accumulation (Figures 3B, 3C and S3A). As previously reported [20], [21], CHK1 was hyperphosphorylated in FANC-deficient cells (Figure 3C). Thus, the deficit in RPA phosphorylation that occurs in these cells cannot merely be ascribed to a defect in ATR activity; it is more likely to represent the consequence of the presence of fewer stretches of ssDNA. In contrast to FANC pathway-deficient cells, MDC1-depleted cells consistently exhibited enhanced levels of p-RPA32 (Figure 3B), increased p-RPA32 focus formation (Figure S3A) and deficits in both H2AX phosphorylation and relocalisation to subnuclear foci (Figures 3B and S3A), as observed in response to IR [39].

These observations support a role for the FANC pathway in preserving DNA integrity by sustaining the formation and maintenance of RPA-coated ssDNA regions and thus limiting DSB formation at stalled forks. MDC1 could be necessary to limit the extension of ssDNA stretches; this might occur through limiting helicase progression and/or by facilitating the formation and repair of stalled replication fork-associated DSBs.

### Crosstalk between the FANC pathway and TLS polymerases at stalled replication forks is necessary for optimal response to UVC exposure

**Figure 4:**
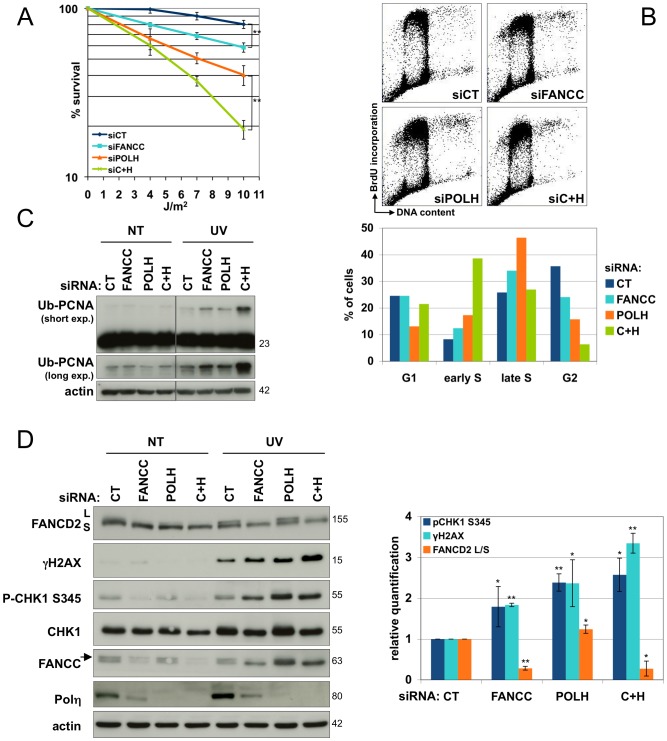
Co-depletion of FANCC and POLH dramatically affects UV response. (**A**) Clonogenic survival of UVC-irradiated cells. HeLa cells transfected with either a nontarget siRNA (CT) or with siRNA targeting FANCC, POLH, or FANCC and POLH (siC+H) were seeded 48 h after transfection and were mock- or UVC-irradiated 6 h later. Surviving colonies were fixed and counted 10 days later. Each data point represents the mean of three independent experiments. ** indicates a significant statistical difference (p<0,01) as calculated using a T-student test. (**B**) Cell cycle analysis of HeLa cells. Cells were mock- or UVC-irradiated (10 J/m^2^) after siRNA transfection and fixed in cold ethanol 24 h later. Cell cycle distribution was revealed by PI/BrdU co-staining and flow cytometry analysis. Each data point represents the mean of two independent experiments. (**C**) PCNA monoubiquitinylation and (**D**) FANCD2 monoubiquitinylation, RPA32 and H2AX phosphorylation analysis. HeLa cells were mock- or UVC-irradiated (10 J/m^2^) 48 h after transfection and harvested 24 h later. The lysates were analyzed by Western blotting using the indicated antibodies. Protein monoubiquitinylation (L/S FANCD2 ratio) and phosphorylation (P-CHK1/total CHK1 and γH2AX/actin) were quantified using Image J software. Each data point represents the mean of three independent experiments. * and ** indicate a significant statistical difference (p<0,05 and p<0,01, respectively) as calculated using a T-student test.

PCNA monoubiquitinylation is required to allow specific and less processive TLS polymerases to drive replication through UVC-damaged DNA. Polη, a member of the Y family of TLS polymerases, is responsible for the timely and error-free bypass of UV-induced lesions [7]. In accord with previous report, Polη depletion is associated with UVC sensitivity (Figure 4A). The simultaneous depletion of Polη and FANCC resulted in increased sensitivity to UVC similar to that observed in NER-deficient cells (LD_50_ = 5 J/m^2^) (compare Figures 4A and 1B). Moreover, the depletion of both Polη and FANCC strongly affected early S-phase cell cycle progression. At 24 h post-UVC exposure, the proportions of wt, FANCC- and Polη-depleted cells in early S-phase were approximately 8%, 12% and 17%, respectively. Interestingly, simultaneous downregulation of FANCC and Polη induced a massive increase in UVC-induced early S-phase cells (39%). Thus, it seems that Polη and the FANC pathway function in parallel and independently to rescue arrested replication machinery.

**Figure 5:**
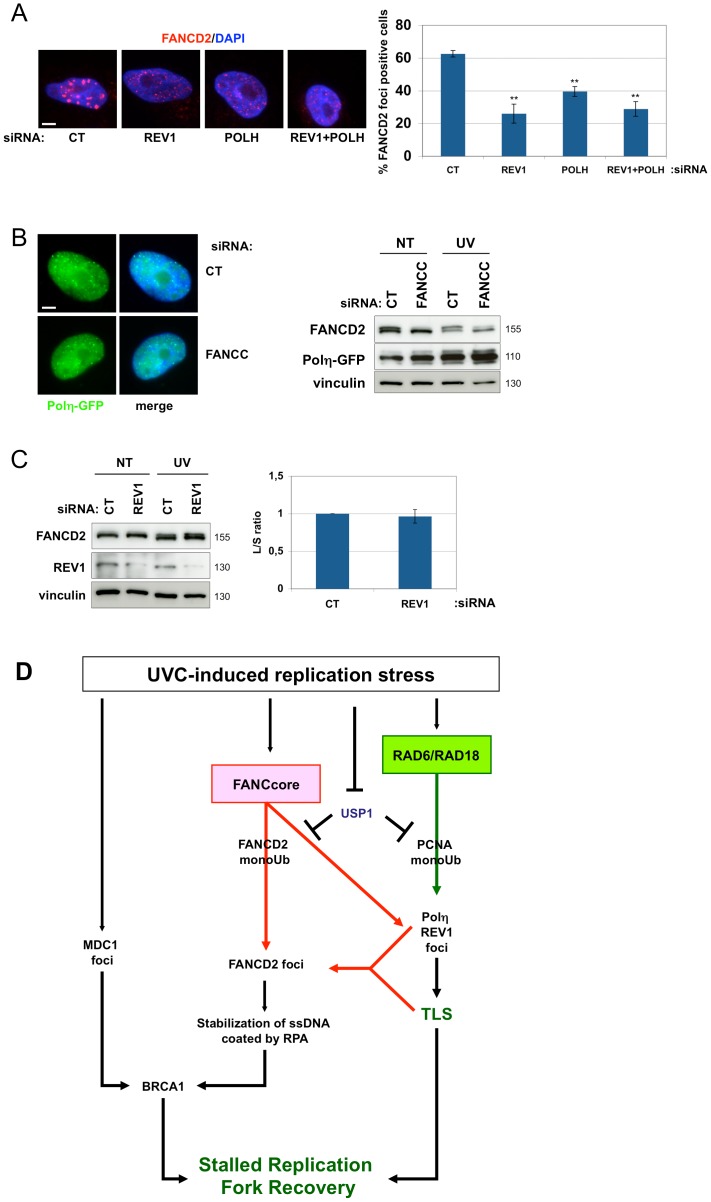
Rev1 and Polη impede optimal localization of FANCD2. (**A**) Immunofluorescence analysis of FANCD2 foci formation. HeLa cells were mock- or UVC-irradiated (10 J/m^2^) 48 h after siRNA transfection with the indicated siRNAs and fixed 24 h later. For foci quantification, cells with more than 5 foci were considered positive. Bar: 5 µm. Each data point represents the mean of three independent experiments. ** indicates a significant statistical difference (p<0,01) as calculated using a T-student test. (**B**) Polh-GFP foci formation. 48 h after transfection with FANCC siRNA, XP30RO-Polh-GFP were irradiated at 10 J/m^2^. 24 h later cells were fixed for a microscope observation (left) or harvested for a Western blot analysis (right). Bar: 5 µm. (**C**) FANCD2 monoubiquitinylation analysis. 48 h after transfection, HeLa cells were irradiated and harvested 24 h later. Lysates were analyzed by Western blotting with the indicated antibodies. The L/S FANCD2 ratio was measured using Image J software. Each data point represents the mean of three independent experiments. (**D**) Model of the replication safeguard mechanisms in UVC-irradiated cells. The model summarizes our observations and integrates published data [31], [52], [59].

The supra-additive effect of loss-of-function of both the FANC pathway and Polη was also observed for UVC-induced Ub-PCNA and γH2AX levels (Figures 4C and 4D). This suggests that inactivation of the FANC pathway and/or Polη results in an accumulation of unresolved stalled replication forks (reflected by the high observed levels of Ub-PCNA) that could progressively collapse and accumulate DSBs, alter chromatin or both (highest levels of γH2AX). Supporting the independent activity of the FANC pathway and Polη, Western blot analysis revealed that the level of UVC-induced FANCD2 monoubiquitinylation was unaffected by Polη depletion (Figure 4D). However, unexpectedly, the frequency and brightness of UVC-induced FANCD2 foci decreased in Polη-deficient and in Polη-depleted cells (Figures 5A and S3B).

Taken together, these observations suggest that loss of function of Polη does not hamper FANC pathway activation and that the TLS step mediated by Polη is required to robustly accumulate FANCD2 on damaged chromatin. Surprisingly, inactivation of the FANC pathway by siRNA-mediated depletion of FANCC resulted in reduced Polη expression (Figure 4D). Likewise, ectopic expression of WT-FANCC in *FANCC-*deficient cells enhanced Polη levels (Figure S4A). Moreover, reduced Polη levels were also observed in siRNA-FANCA-depleted but not in siRNA-FANCD2-depleted cells (Figure S4B and S4C), suggesting the exclusive involvement of the FANC core complex in Polη expression/stabilisation. Unfortunately, we could not determine whether or to what extent reduced expression of Polη impacted the capability of the TLS polymerase to relocalise and accumulate in nuclear foci. Indeed, antibodies directed against Polη gave poor results for the detection of Polη by immunofluorescence, and FANCC depletion in a Polη-GFP overexpressing cellular model [40] failed to reduce the level of exogenously overexpressed tagged protein (Figure 5B).

### Rev1 is also involved in the optimal relocalisation of FANCD2

Rev1 is another member of the Y family of polymerases that can interact with Ub-PCNA. Rev1 relocalises to Ub-PCNA simultaneously with but independently of Polη [41] and is required for the recruitment of the Rev3/Rev7 dimer (Polζ) to Ub-PCNA foci [42]. According to the two-step TLS model, Polζ is involved in nascent DNA elongation after the intervention of other Y-family polymerases, and it suppresses chromosome abnormalities and participates in TLS in XP-V cells [7].

Recently, the FANC core complex has been linked to optimal Rev1 relocalisation and activity in response to UVC lesions [22], [43]. However, the converse, i.e., the involvement of Rev1 in FANC pathway activity/activation, has not been explored. Similar to the absence of Polη, siRNA-mediated downregulation of Rev1 did not modify FANCD2 monoubiquitinylation (Figure 5C); however, Rev1 downregulation significantly hampered the relocalisation of FANCD2 to nuclear foci (Figure 5A). Finally, simultaneous depletion of Polη and Rev1 did not exacerbate the disruption of FANCD2 relocalisation to nuclear foci (Figure 5A). It is interesting to note that TLS polymerase-depleted HeLa and U2OS cells displayed weaker FANCD2 foci than control cells (Figures 5A and S5C).

Taken together, these results establish the existence of a connection between the FANC pathway and the TLS process. The FANC core complex appears to regulate the level of Polη and the relocalisation of Rev1, and both are required for the optimal relocalisation of Ub-FANCD2.

## Discussion

In DDR/DDT-proficient cells, the NER pathway requires more than 48 h to fully eliminate UVC-induced photolesions. Thus, ongoing replication forks are inevitably arrested as they meet unrepaired lesions. This creates a critical situation that the cell must resolve to limit genetic instability, which is one of the main causes and hallmarks of cancer [1]. As a consequence of polymerase/helicase uncoupling, regions containing ssDNA form beyond the lesion that blocks replication. These ssDNA regions are a substrate for the aggregation of an RPA filament that protects ssDNA from cleavage and degradation and that is necessary, together with FANCM/FAAP24 [44], [45], for the full activation of the ATR-signalling pathway [46], which is required to recover stalled replication forks.

Our results provide new insights into the protein network involved in rescuing UVC-stalled replication forks (Figure 5D). A stalled fork triggers at least two monoubiquitinylation pathways, the RAD6/RAD18-mediated monoubiquitinylation of PCNA and the FANC core complex-mediated monoubiquitinylation of FANCD2 [47]. Ub-PCNA is required for the optimal relocalisation of Polη and Rev1 to UVC stalled replication forks for TLS [7], whereas Ub-FANCD2 is required for the optimal relocalisation of RAD51 and execution of HR, at least after ICLs formation [18], [25]. It has been reported that defects in the RAD6/RAD18-PCNA pathway impact optimal FANCD2 monoubiquitinylation and/or relocalisation to nuclear foci [48], [49]. Here, we have extended the above connection by demonstrating that depletion of Polη or Rev1 alone is sufficient to alter the ability of Ub-FANCD2 to assemble in nuclear foci. Thus, it appears that the loading of FANCD2 into nuclear foci requires not only full monoubiquitinylation but also a lesion-bypass step mediated by Polη and/or Rev1. Consequently, the previously described alteration of FANCD2 relocalisation in cells with an altered RAD6/RAD18-PCNA pathway [48], [49] appears to be linked to the disrupted localisation of Polη and Rev1. It is noteworthy that the FANC core complex, but not FANCD2, is, in turn, required for the maintenance of Polη levels (this work) and Rev1 relocalisation [22]. As further evidence of the tight and finely tuned connections between these two monoubiquitinylation pathways, it is important to note that both are simultaneously shut down by the same deubiquitinase, USP1, the expression of which is rapidly downregulated after UVC exposure [50], [51], permitting the full ubiquitinylation of PCNA and FANCD2.

Loss of function of Polη has been associated with RPA and CHK1 hyperphosphorylation [52], whereas FANC pathway disruption enhances CHK1 and H2AX phosphorylation [21] but decreases p-RPA32 levels (Figure 3B). These data indicate that TLS reduces the formation of long RPA-coated ssDNA stretches, whereas FANCD2 appears to be necessary for stabilising RPA-coated ssDNA regions to reduce the risk of forming DSBs. It has recently been reported that BRCA1 depletion in response to UVC is associated with a decrease in RPA-coated ssDNA regions and with hypophosphorylation of both RPA and CHK1 [34]. Thus, downstream of ATR, BRCA1 appears to be involved in both checkpoint activation and the efficient recovery of stalled replication forks, whereas, because the CHK1–dependent checkpoint is hyperactivated in FA-deficient cells, FANCD2 appears to be required only for the latter process [21].

Interestingly, FANCD2 and BRCA1 relocalise soon after UVC irradiation in a partially mutually dependent manner. BRCA1 is involved in the relocalisation of the RFC and 9-1-1 complexes to UVC-damaged DNA [34]. These complexes, in turn, promote optimal FANCD2 monoubiquitinylation and assembly in nuclear foci [20], thus explaining the less efficient relocalisation of FANCD2 in BRCA1-deficient cells [19]. Early BRCA1 relocalisation to UVC-damaged DNA depends both on the FANC pathway and on MDC1, and the latter is also necessary for subsequent BRCA1 focalisation. How FANCC and FANCD2 are involved in optimal BRCA1 relocalisation in UVC-challenged cells remains to be determined. By contrast, BRCA1-relocalisation defects in MDC1-deficient cells are likely due to a lack of MDC1-mediated histone post-translational modifications necessary for BRCA1 assembly. Because FANCD2 and MDC1 relocalise to UVC-damaged areas independently and with identical kinetics, their different effects on BRCA1 confirm that MDC1 associates with stalled replication forks before DNA breakage [32]. Moreover, MDC1 acts in parallel with FANC proteins to recruit BRCA1 to ssDNA regions to promote replication recovery by TS, an HR-driven mechanism, without the formation of DSBs. Later, during recovery from DSBs associated with stalled replication forks, BRCA1 relocalisation is solely controlled by MDC1, as has been described for the cellular response to IR-induced DSBs [53].

It is interesting to note that several of the proteins that are analysed and discussed in the current study are involved in RAD51 relocalisation and activity. In particular, previous evidence have suggested that Polη may be required for 3′-repriming after RAD51-mediated D-loop formation (a D-loop is a structure created by strand invasion either during TS or during DSB repair) [54], [55]. Polη- or Rev1–dependent FANCD2 relocalisation could be necessary to mediate RAD51 loading and D-loop formation after TLS to fill the ssDNA gap that occurs behind an arrested replication fork. This would enable the use of Polη to initiate replication after strand invasion and to close the gap by rapidly reducing ssDNA extension and/or impeding DSB formation.

It is likely that when DNA lesions impede replication fork progression, cells use TLS and TS as the first line of defence to complete DNA replication and preserve genome stability. TLS and TS are intended to safeguard the structural integrity of DNA. Prolonged stalling or lack of proper stabilisation of a stalled fork/ssDNA can lead to DNA breakage that necessitates BIR, a process that requires BRCA1 to load proteins such as CtIP and FANCJ to mediate DSB resectioning. Indeed, Polη- Rev1-, BRCA1- and FANC-pathway-deficient cells are all characterised by high levels of sister chromatid exchange and/or complex chromosome rearrangements [56], [57], consistent with the disruption of HR in response to DNA damage. Thus, when cells fail to properly bypass fork-blocking lesions using TLS or TS, BIR could occur, leading to increased genetic instability.

## Materials and Methods

### Cell culture and treatments

HeLa and U2OS cells as well as SV40-immortalized MRC5 fibroblasts (ATCC); SV40-immortalized XP fibroblasts XP12RO (XP-A, a gift of Dr Alain Sarasin, UMR8200 CNRS, Institut Gustave Roussy, Villejuif, France. Bootsma et al., Mutat. Res., 9, 507-516, 1970); GM8437 (XP-F, obtained from the Coriell Institute, Camden, New Jersey, USA) and XP30RO (XP-V); XP30RO (XP-V); XP30ROpolh (XP-V stably expressing POLH cDNA) or XP30RO-GFP-polh (XP-V-POLh-GFP) (were a gift of Dr Patricia Kannouche, UMR8200 CNRS, Institut Gustave Roussy, Villejuif, France; Kannouche et al. Genes Dev., 15; 158–172, 2001); SV40-immortalized FA fibroblasts PD331 (FA-C, *FANCC* vector-transduced +EV or FANCC-corrected +*FANCC*,) and PD352 (FA-G. All FA cells were from the Fanconi Anemia Research Found, Eugene, OR, USA); normal human primary fibroblasts AS911 (The cell line was part of a diagnosis effort on DNA repair diseases in the framework of the European Community contract ^3^GENESKIN^2^,LSHM-CT-2005-512117. The laboratory has the approval to derive, to study and to stock cell lines with the agreement of the French Ministry of Health, and approval of the CNRS. All participants (or guardians of minors) signed a written informed consent. The sample was provided anonymously). All cell lines were routinely grown in DMEM (Gibco) supplemented with 10% foetal calf serum (Dutcher), 1 mM sodium pyruvate, 100 U/mL penicillin and 100 µg/mL streptomycin (all from Gibco) at 37°C in 5% CO_2_. XP30ROpolh and PD331 cells were grown in the presence of 100 mg/ml zeocin (Invitrogen) and 0.5 g/mL puromycin, respectively. When necessary, BrdU or EdU (Invitrogen) was added to the medium at a final concentration of 10 µM.

For UVC irradiation (254 nm), cells were washed in phosphate-buffered saline (PBS) and irradiated at a fluency of 0.24 or 0.46 J/m^2^/s. Localised UVC irradiation was performed as previously described using a 5-µm polycarbonate filter (Millipore) [58].

For clonogenic survival, 500–1000 cells per 6-cm diameter plate were plated 48 h after transfection and 6 h before mock- or UVC-irradiation. Ten days later, the clones were stained with methylene blue and counted. Each data point represents the mean of three independent experiments.

### Transfection

Single siRNAs were purchased from Eurogentec. The following sequences were used: Ctrl (CT, untargeted) (CGUCGACGGAAUACUUCGATT), FANCD2 (GGAGAUUGAUGGUCUACUA), MDC1a (GUCUCCCAGAAGACAGUG) and MDC1b (ACAGUUGUCCCCACAGCCC) used as a mix and POLH (GAAGUUAUGUCCAGAUCUU). FANCC smartpool siRNA was obtained from Dharmacon. Rev1 siRNA was also purchased from Dharmacon (D3: GAAAUGUGCUGCCUCUGUU). Transfection of siRNA (20 nM) was performed using Interferin (PolyPlus transfection), and experiments were carried out 24 to 48 h later. In the case of double transfection, the same total amount of siRNA was used, adding control siRNA to the single siRNA of interest.

### Western blotting

Collected cells were disrupted in lysis buffer (50 mM Tris pH 7.9, 150 mM NaCl, 1 mM EDTA, 0.5% NP-40 supplemented with protease and phosphatase inhibitors (Roche)). After a 20-min incubation on ice, the lysates were sonicated, combined with 4X Laemmli buffer containing β-2-mercaptoethanol and denatured by boiling. Proteins (25 µg) were separated by SDS-PAGE. The antibodies used were directed against FANCD2 (FI-17), CHK1 (G-4), actin (I-19), PCNA (PC-10), MDC1 (ab11169, Abcam), Polη (ab17725, Abcam), vinculin (ab18058, Abcam), p345CHK1 (2348, Cell Signaling), RPA32 (NA19L, Calbiochem), p-RPA32 S33 (A300-246A, Bethyl) and γH2AX (JBW301, Upstate). The Rev1 antibody was kindly provided by P. Kannouche's laboratory.

### Immunofluorescence

Cells grown on glass coverslips were fixed in 4% formaldehyde for 10 min at room temperature before permeabilisation in 0.5% Triton X-100 for 5 min. For some experiments, cells were pre-extracted in CSK100 buffer (10 mM PIPES pH 6.8, 100 mM NaCl, 3 mM MgCl_2_, 1 mM EGTA, 300 mM sucrose, 0.2% Triton X100) prior to fixation. After blocking with 3% BSA in PBS containing 0.05% Tween 20, the cells were stained for 1 h in blocking solution with antibodies against FANCD2 (Abcam ab2187, 1/1000), MDC1 (Abcam ab11169, 1/300), ERCC1 (Santa Cruz Biotechnology D-10 or FL-297, 1/100), γH2AX (Upstate JBW301, 1/5000), CPD (Cosmobio TDM-2, 1/1000), 6,4-PP (Cosmobio 64M-2, 1/300), BrdU (Abcam ab6326, 1/250), BRCA1 (Calbiochem OP92, 1/50), 53BP1 (Millipore MAB3802, 1/500) or p-RPA32 S33 (Bethyl A300-246A, 1/3000). Primary antibody detection was achieved by incubation with anti-rabbit, anti-goat or anti-mouse Alexa Fluor 315, 488 or 594-conjugated secondary antibodies (Invitrogen; 1/1000) for 30 min at room temperature. Slides were mounted in DAKO mounting medium supplemented with DAPI (Sigma) and examined at a magnification of 63× by fluorescence microscopy (Zeiss Axio Observer Z1). Images were captured with an ORCA-ER camera (Hamamatsu). The microscope and camera parameters were adjusted for each series of experiments to avoid signal saturation. Image processing and analysis were performed using Image J software (http://rsb.info.nih.gov/ij/).

For CPD, 6,4-PP and BrdU detection, DNA was denatured in 2 N HCl for 10 min at 37°C prior to blocking and staining. EdU detection was performed according to the manufacturer's instructions (Invitrogen).

### Flow cytometry

Cells were irradiated and incubated for 24 h. At 10 min prior to harvesting, 10 µM BrdU was added to the culture medium. The cells were trypsinised, washed in PBS and fixed in 80% ice-cold ethanol at −20°C until use. The samples were then incubated in 15 mM pepsin for 20 min at 37°C, and the DNA was denatured in 2 N HCl for 20 min at room temperature. The pellet was washed in Bu buffer (0.1 M Hepes in PBS, 0.1% FCS, 0.1% Tween 20) and incubated with 1/50 mouse anti-BrdU antibody (DAKO) in Bu buffer for 45 min at room temperature. The samples were then incubated with 1/100 Alexa 488-conjugated donkey anti-rabbit secondary antibody (Invitrogen). Cellular DNA was stained with 25 mg/mL propidium iodide and 50 mg/ml RNAse A (Sigma) in PBS for 30 min at room temperature prior to analysis. The samples were analysed with a C6 flow cytometer (Accuri).

## Supporting Information

Figure S1 **UV irradiation activates the FANC pathway in S-phase and independently of the Nucleotide Excision Repair pathway.** (**A**) FANCD2 (red) co-localization with CPD (green) in S-phase cells. HeLa cells were locally UVC-irradiated (100 J/m^2^) and fixed 2 h later. BrdU (blue) was added 10 min before fixation. Bar: 20 µm. (**B**) 6,4-PPs and CPDs removal quantification. Wt MRC5, FA-G and XP-F cells were locally UVC-irradiated (100 J/m^2^) and fixed in formaldehyde at the indicated times. Both lesions were stained using suitable antibody and around 100 cells were scored. Each data point represents the mean of two independent experiments. (**C**) Immunofluorescence analysis of CPD (green)/ERCC1 (red) or FANCD2 co-localization in S-phase/BrdU MRC5 cells. Cells were locally UVC-irradiated (100 J/m^2^) and fixed at the indicated times. Bar:10 µm. (**D**) ERCC1 recruitment in FA-G cells. The cells were treated as in (C) and fixed 1 h later. Bar: 10 µm. (**E**) L/S FANCD2 relative ratio quantification from figure 1E. The ratio was measured using Image J software. Each data point represents the mean of two independent experiments. (**F**) FANCD2 recruitment in FA-G, XP-A and XP-F cells. The cells were treated as in (C) and fixed 1 h later. Bar: 10 µm.
(TIF)

Figure S2 **MDC1 and FANCD2 connection in response to UVC exposure.** (**A**) *Left*, immunofluorescence analysis of CPD (blue)/MDC1 (red)/EdU (green) co-localization in MRC5 cells. Cells were locally UVC-irradiated (100 J/m^2^) and fixed at the indicated times. EdU was added 10 min before fixation. *Right*, Recruitment kinetics of MDC1. Wt MRC5, XP and FA fibroblasts were locally UVC-irradiated (100 J/m^2^) and fixed at different times later. SiRNA-transfected MRC5 cells were irradiated 48 h after transfection. Co-localization between ERCC1 and CPDs was quantified in at least 100 cells per time point. Bar:10 µm. (**B**) FANCD2 monoubiquitinylation analysis. 48 h after transfection, PD331 FA-C (+EV, empty vector) or complemented (+FANCC) cells were mock- or UVC-irradiated (10 J/m^2^) and harvested 4 h later. The lysates were analyzed by Western blotting using the indicated antibodies. L/S FANCD2 relative ratio was measured using Image J software. Each data point represents the mean of three independent experiments. * and ** indicate a significant statistical difference (p<0,05 and p<0,01, respectively) as calculated using a T-student test. (**C**) FANCD2 foci number quantification in UVC-irradiated cells from figure 2D. (**D**) MDC1 foci formation quantification. PD331 (+EV or +FANCC) cells were mock- or UVC-irradiated (10 J/m^2^) and fixed 24 h later. Cells with more than 5 foci were considered as positive. Each data point represents the mean of three independent experiments. * indicates a significant statistical difference (p<0,05) as calculated using a T-student test.
(TIF)

Figure S3 **FANC pathway prevents DSB formation and favors the maintenance of ssDNA regions.** (**A**) Immunofluorescence analysis of p-RPA32 and γH2AX foci formation. HeLa cells were mock- or UVC-irradiated (10 J/m^2^) 48 h after transfection and fixed 4 or 24 h later. For foci quantification, cells with more than 5 foci were considered as positive. Each data point represents the mean of three independent experiments. * and ** indicate a significant statistical difference (p<0,05 and p<0,01, respectively) as calculated using a T-student test. (**B**) Immunofluorescence analysis of FANCD2/γH2AX foci formation. MRC5, XP30RO XP-V and XP30RO Cl6 complemented cells were mock- or UVC-irradiated (10 J/m^2^) and fixed 24 h later. Bars: 5 µm.
(TIF)

Figure S4 **FANCcore complex proteins regulate Polη.** Polη expression analysis by Western blotting in PD331 (+EV and + FANCC) (**A**) and siRNA-transfected HeLa (**B and C**) cells harvested before treatment or 24 h after UVC irradiation (10 J/m^2^). Asterisk: aspecific band.
(TIF)

Figure S5 **Validation in U2OS cells of previous data obtained in HeLa and human fibroblasts.** Some experiments presented in figure 2C, 2D and 5A, respectively, were repeated in U2OS cells. (**A**) FANCD2 monoubiquitinylation analysis. U2OS cells were mock- or UVC-irradiated (10 J/m^2^) 48 h after transfection and harvested 4 h later. The lysates were analyzed by Western blotting using the indicated antibodies. The Long form (monoubiquitinylnated form)/Short form relative ratio (L/S ratio) was measured using Image J software. (**B**) Immunofluorescence analysis of FANCD2 focus formation in MDC1-depleted cells. U2OS cells were mock- or UVC-irradiated (10 J/m^2^) 48 h after transfection with untargeted or a MDC1-targeted siRNA. 24 h later, they were prepermeabilized then fixed. For foci-positive cell quantification, cells with more than 5 foci were counted. (**C**) Immunofluorescence analysis of FANCD2 foci formation. U2OS cells were mock- or UVC-irradiated (10 J/m^2^) 48 h after siRNA transfection with the indicated siRNAs and fixed 24 h later. For foci quantification, cells with more than 5 foci were considered positive.
(TIF)
