# Supplementary figures and images for: FANC Pathway Promotes UV-Induced Stalled Replication Forks Recovery by Acting Both Upstream and Downstream Polη and Rev1
Source: PLoS One. 2013 Jan 24;8(1):e53693. doi: 10.1371/journal.pone.0053693 (PMC3554758; doi:10.1371/journal.pone.0053693)

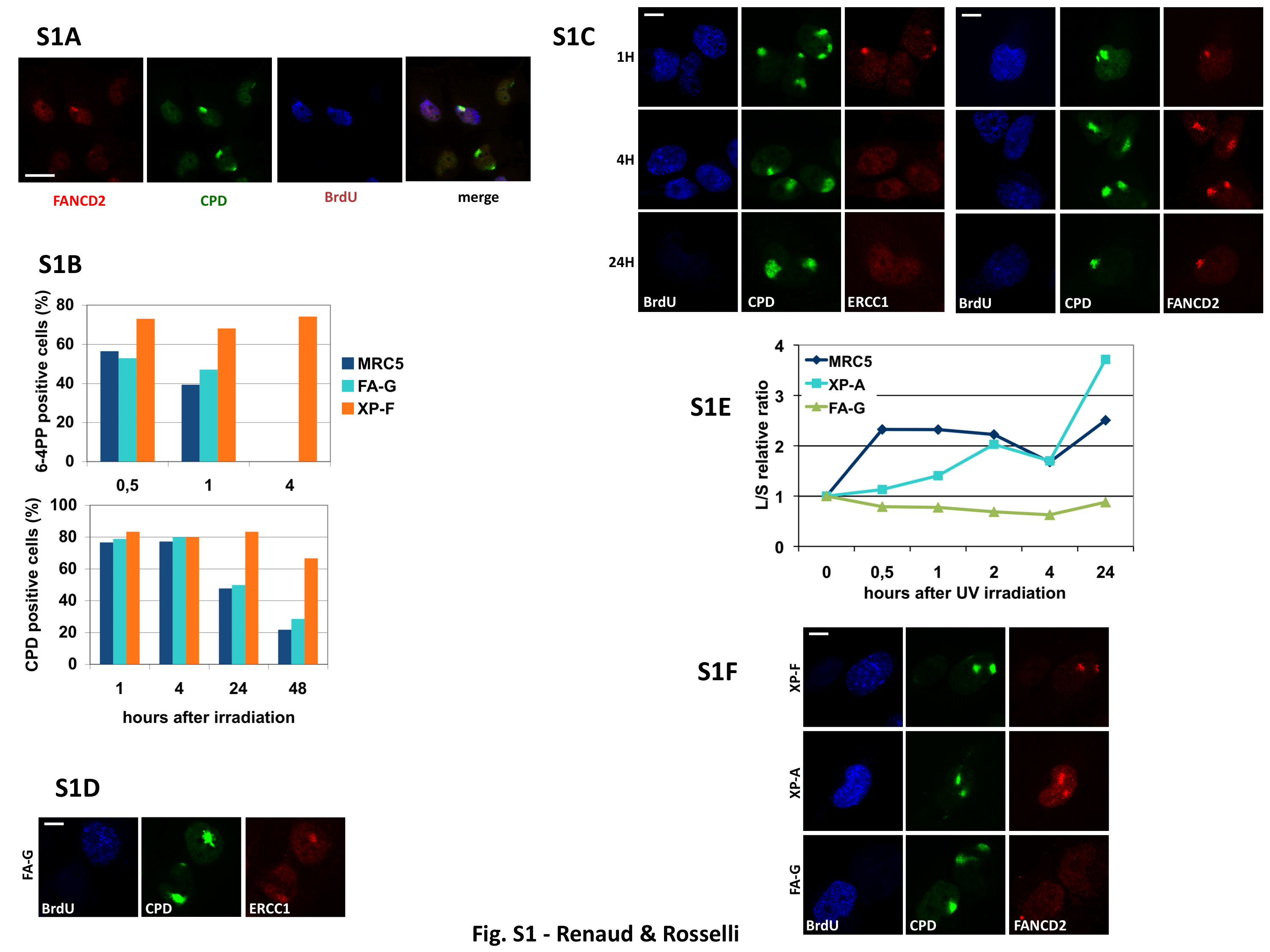

Supplement: Figure S1 — UV irradiation activates the FANC pathway in S-phase and independently of the Nucleotide Excision Repair pathway. (A) FANCD2 (red) co-localization with CPD (green) in S-phase cells. HeLa cells were locally UVC-irradiated (100 J/m2) and fixed 2 h later. BrdU (blue) was added 10 min before fixation. Bar: 20 µm. (B) 6,4-PPs and CPDs removal quantification. Wt MRC5, FA-G and XP-F cells were locally UVC-irradiated (100 J/m2) and fixed in formaldehyde at the indicated times. Both lesions were stained using suitable antibody and around 100 cells were scored. Each data point represents the mean of two independent experiments. (C) Immunofluorescence analysis of CPD (green)/ERCC1 (red) or FANCD2 co-localization in S-phase/BrdU MRC5 cells. Cells were locally UVC-irradiated (100 J/m2) and fixed at the indicated times. Bar:10 µm. (D) ERCC1 recruitment in FA-G cells. The cells were treated as in (C) and fixed 1 h later. Bar: 10 µm. (E) L/S FANCD2 relative ratio quantification from figure 1E. The ratio was measured using Image J software. Each data point represents the mean of two independent experiments. (F) FANCD2 recruitment in FA-G, XP-A and XP-F cells. The cells were treated as in (C) and fixed 1 h later. Bar: 10 µm. (TIF) [file pone.0053693.s001.tif]

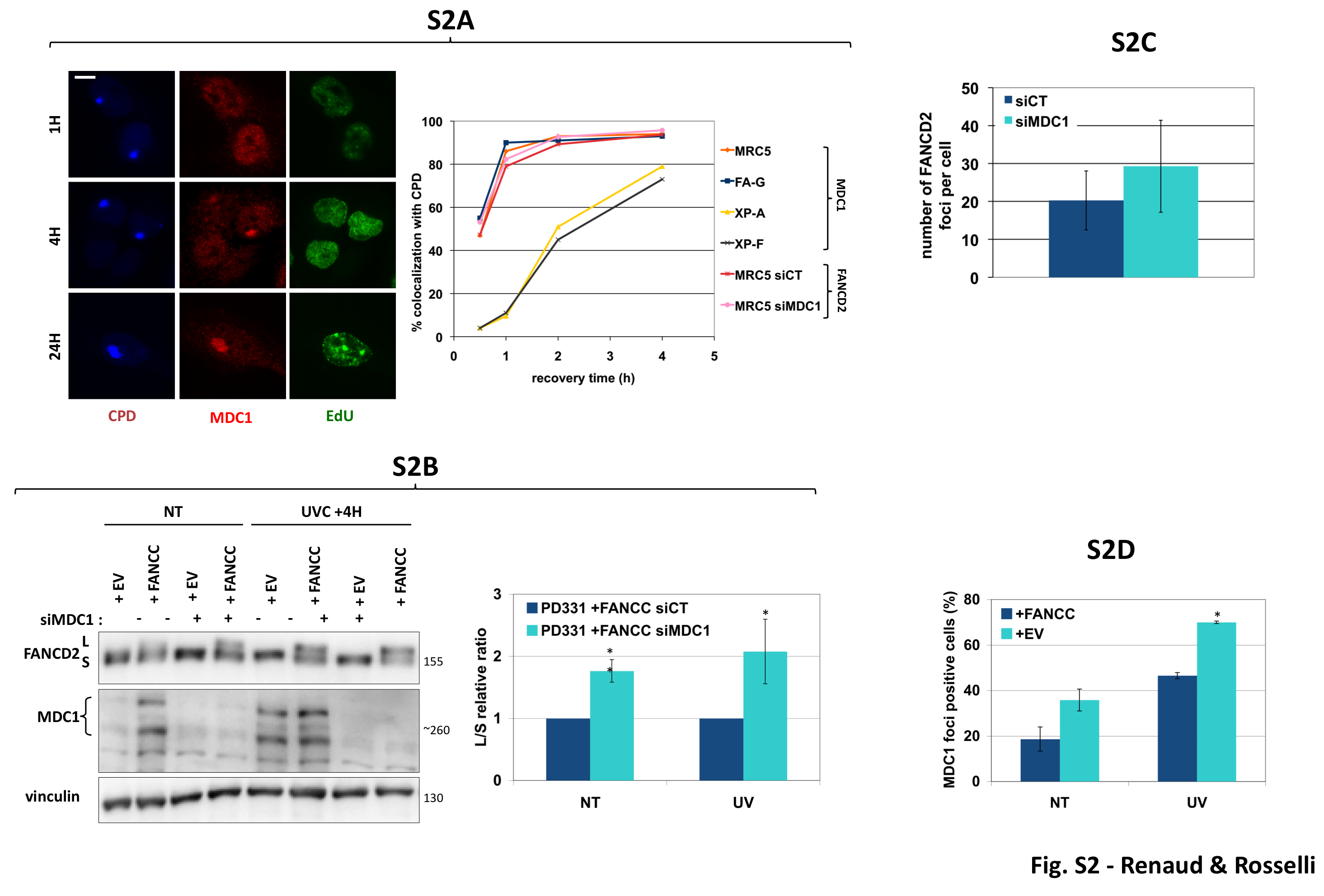

Supplement: Figure S2 — MDC1 and FANCD2 connection in response to UVC exposure. (A) Left, immunofluorescence analysis of CPD (blue)/MDC1 (red)/EdU (green) co-localization in MRC5 cells. Cells were locally UVC-irradiated (100 J/m2) and fixed at the indicated times. EdU was added 10 min before fixation. Right, Recruitment kinetics of MDC1. Wt MRC5, XP and FA fibroblasts were locally UVC-irradiated (100 J/m2) and fixed at different times later. SiRNA-transfected MRC5 cells were irradiated 48 h after transfection. Co-localization between ERCC1 and CPDs was quantified in at least 100 cells per time point. Bar:10 µm. (B) FANCD2 monoubiquitinylation analysis. 48 h after transfection, PD331 FA-C (+EV, empty vector) or complemented (+FANCC) cells were mock- or UVC-irradiated (10 J/m2) and harvested 4 h later. The lysates were analyzed by Western blotting using the indicated antibodies. L/S FANCD2 relative ratio was measured using Image J software. Each data point represents the mean of three independent experiments. * and ** indicate a significant statistical difference (p<0,05 and p<0,01, respectively) as calculated using a T-student test. (C) FANCD2 foci number quantification in UVC-irradiated cells from figure 2D. (D) MDC1 foci formation quantification. PD331 (+EV or +FANCC) cells were mock- or UVC-irradiated (10 J/m2) and fixed 24 h later. Cells with more than 5 foci were considered as positive. Each data point represents the mean of three independent experiments. * indicates a significant statistical difference (p<0,05) as calculated using a T-student test. (TIF) [file pone.0053693.s002.tif]

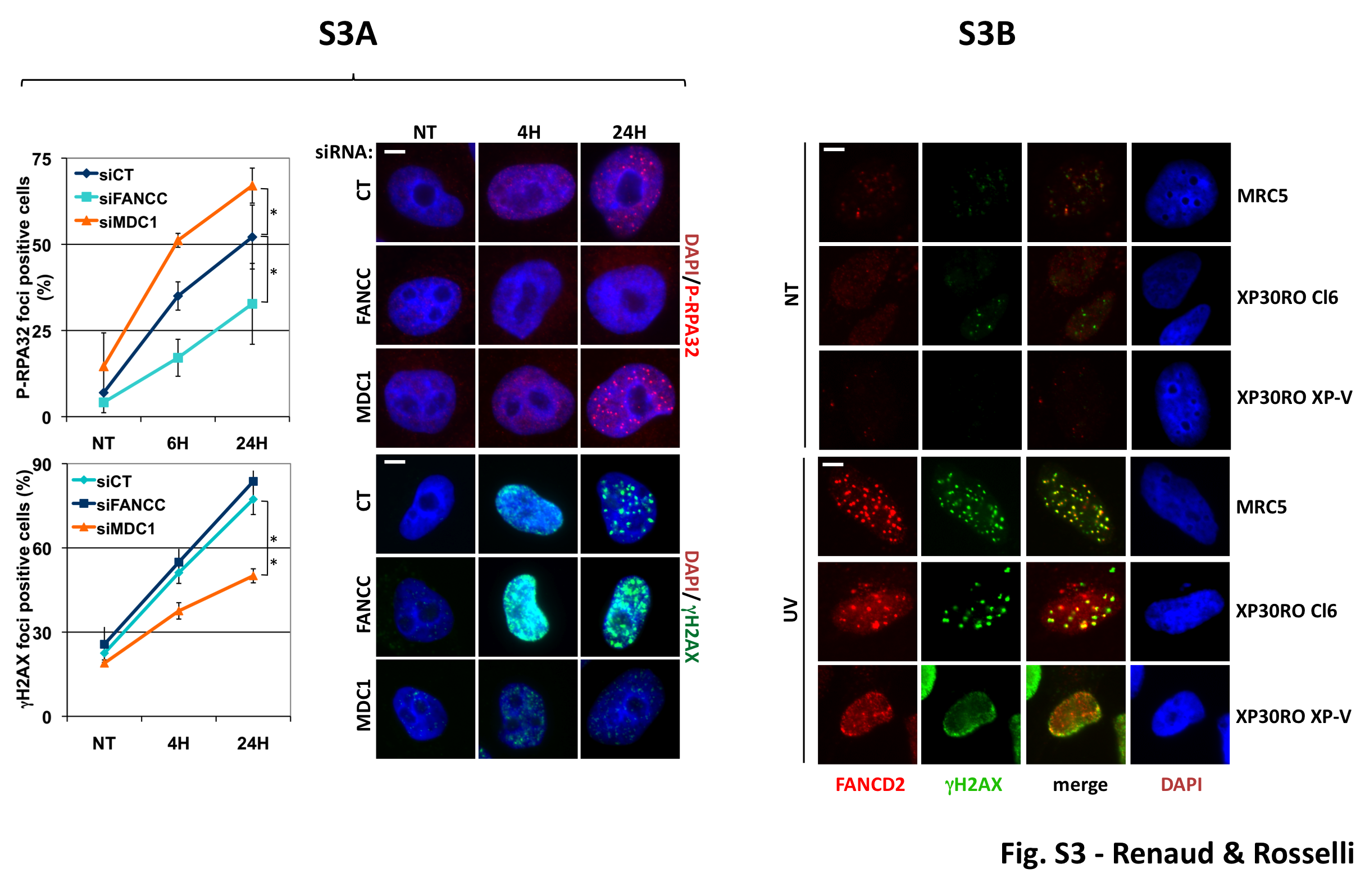

Supplement: Figure S3 — FANC pathway prevents DSB formation and favors the maintenance of ssDNA regions. (A) Immunofluorescence analysis of p-RPA32 and γH2AX foci formation. HeLa cells were mock- or UVC-irradiated (10 J/m2) 48 h after transfection and fixed 4 or 24 h later. For foci quantification, cells with more than 5 foci were considered as positive. Each data point represents the mean of three independent experiments. * and ** indicate a significant statistical difference (p<0,05 and p<0,01, respectively) as calculated using a T-student test. (B) Immunofluorescence analysis of FANCD2/γH2AX foci formation. MRC5, XP30RO XP-V and XP30RO Cl6 complemented cells were mock- or UVC-irradiated (10 J/m2) and fixed 24 h later. Bars: 5 µm. (TIF) [file pone.0053693.s003.tif]

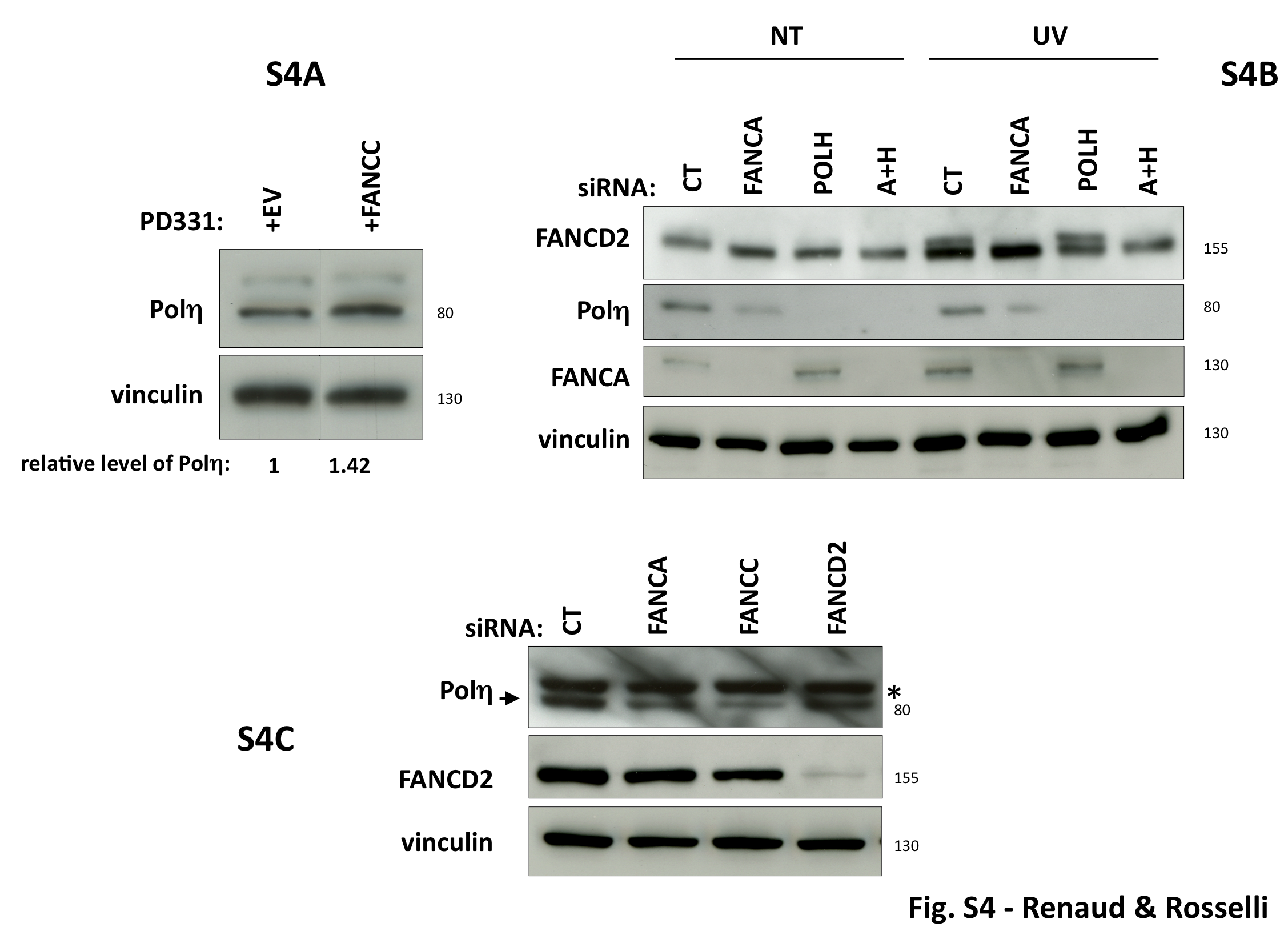

Supplement: Figure S4 — FANCcore complex proteins regulate Polη. Polη expression analysis by Western blotting in PD331 (+EV and + FANCC) (A) and siRNA-transfected HeLa (B and C) cells harvested before treatment or 24 h after UVC irradiation (10 J/m2). Asterisk: aspecific band. (TIF) [file pone.0053693.s004.tif]

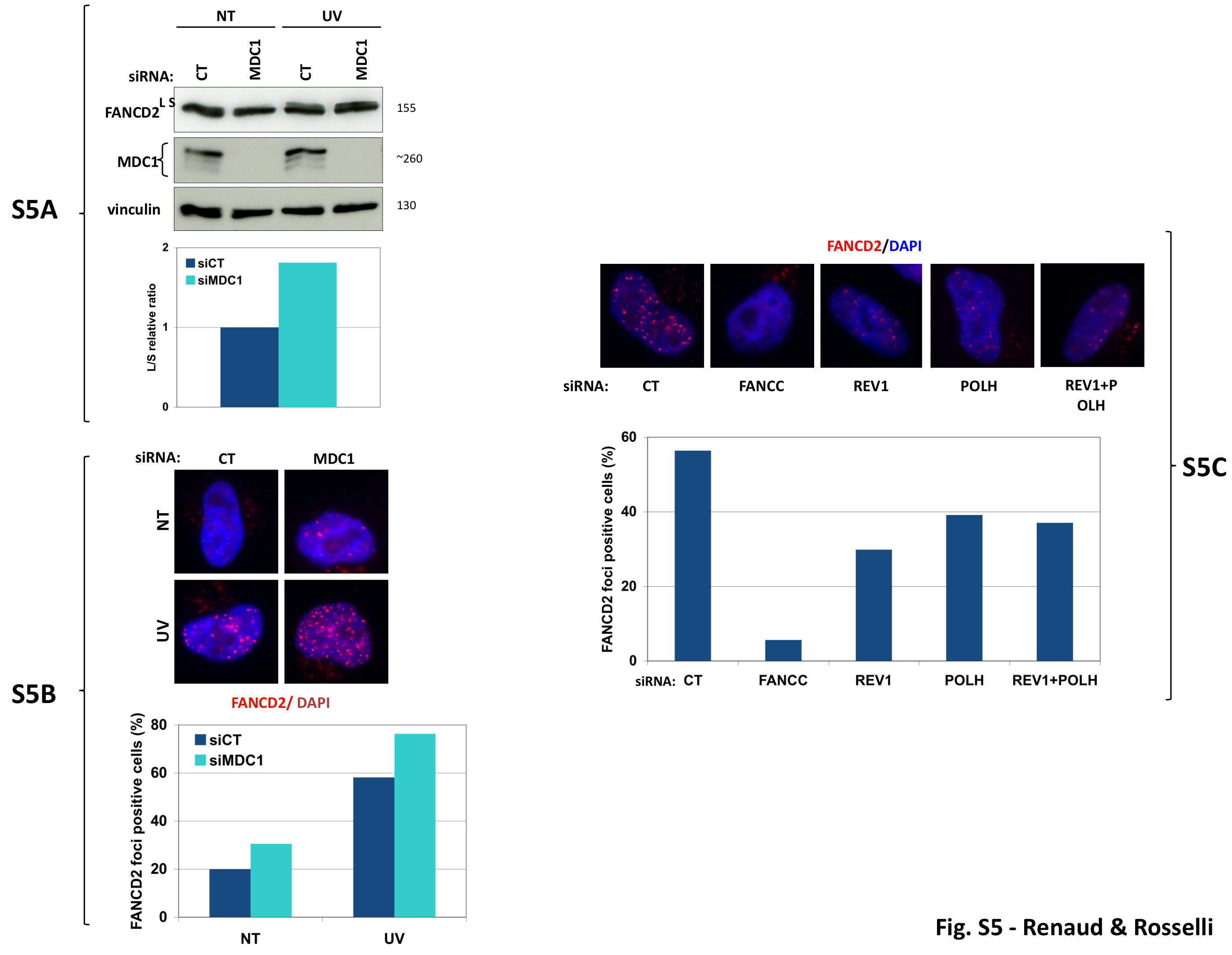

Supplement: Figure S5 — Validation in U2OS cells of previous data obtained in HeLa and human fibroblasts. Some experiments presented in figure 2C, 2D and 5A, respectively, were repeated in U2OS cells. (A) FANCD2 monoubiquitinylation analysis. U2OS cells were mock- or UVC-irradiated (10 J/m2) 48 h after transfection and harvested 4 h later. The lysates were analyzed by Western blotting using the indicated antibodies. The Long form (monoubiquitinylnated form)/Short form relative ratio (L/S ratio) was measured using Image J software. (B) Immunofluorescence analysis of FANCD2 focus formation in MDC1-depleted cells. U2OS cells were mock- or UVC-irradiated (10 J/m2) 48 h after transfection with untargeted or a MDC1-targeted siRNA. 24 h later, they were prepermeabilized then fixed. For foci-positive cell quantification, cells with more than 5 foci were counted. (C) Immunofluorescence analysis of FANCD2 foci formation. U2OS cells were mock- or UVC-irradiated (10 J/m2) 48 h after siRNA transfection with the indicated siRNAs and fixed 24 h later. For foci quantification, cells with more than 5 foci were considered positive. (TIF) [file pone.0053693.s005.tif]
